# The Dental Management of Pediatric Patient Diagnosed with Myasthenia Gravis: A Case Report

**DOI:** 10.1055/s-0042-1745773

**Published:** 2022-06-21

**Authors:** Saad M. AlManea, Mashael A. AlHadlaq, Noura M. AlBuqmi, Sultan S. AlGomaiz

**Affiliations:** 1 Department of Pediatric Dentistry, Ministry of National Guard - Health Affairs, Riyadh, Saudi Arabia; 2 Department of Dental Services, Ministry of National Guard - Health Affairs, Riyadh, Saudi Arabia

**Keywords:** myasthenia gravis, pediatric, dental management, dentistry, dental rehabilitation

## Abstract

Myasthenia gravis (MG) is a rare autoimmune neurological disorder characterized by muscle weakness and can vary in severity from the mild form that affects the ocular muscles only to the generalized form that affects the other muscle groups. The prevalence of MG is 150 to 200 per million population over the past 50 years, and approximately 10% of these cases are pediatric patients where the disease onset starts before the age of 18 years. The etiology of MG is due to the blockage of neuromuscular transmission by circulating autoantibodies targeting mainly the nicotinic acetylcholine receptor (AChR) and associated proteins in the postsynaptic membrane of skeletal muscles. In MG patients, dental treatment is challenging due to the nature of the condition and its complexity. Moreover, dentists treating MG patients should acquire knowledge about the disease background and the special considerations that need to be taken. In this case report, our aim was to raise awareness about MG among dentists and discuss the dental management of the patients who have this disease and the precautions that should be taken. This case report presents a pediatric MG patient with poor oral hygiene, multiple decayed teeth, dental fluorosis, tongue thrust oral habit that led to anterior open-bite and uncomplicated crown fracture of the upper permanent central incisors. The decision was made to treat the patient under general anesthesia because of the medical condition and the extent of the dental treatment. In the process of preparing the patient for surgery, the patient was cleared from the treating physicians and was admitted under neurology care as per the primary physician. On the day of surgery, the patient received full-mouth dental rehabilitation under general anesthesia, including pulp therapy, crowns, restorations, and extractions. Furthermore, she was seen regularly in recall visits every 3 months.

## Introduction

Myasthenia gravis (MG) is a rare autoimmune disorder characterized by muscle weakness, and it is caused by blockage of neuromuscular transmission due to circulating autoantibodies, targeting mainly the nicotinic acetylcholine receptor (AChR) and associated protein in the postsynaptic membrane of skeletal muscles. 1 The muscle weakness varies in severity from mild weakness that only involves the ocular muscles to generalized muscle weakness. In fact, the initial presentation in 85% of cases is weakness of the ocular muscle characterized by ptosis and/or diplopia. 1 The prevalence of MG is 150 to 200 per million population over the past 50 years, and the incidence rate is 4.1 to 30 cases per million person-years. However, approximately 10% of the total cases are pediatric patients whose disease onset starts before the age of 18 years. In fact, pediatric MG is considered even rarer with an incidence rate of 1 to 5 cases per million person-years. Unfortunately, there is sparse evidence of MG in Saudi Arabia and it has not been studied extensively. 2 Al-Moallem et al and AlAnazy studied the clinical profile of MG in Saudi Arabia and reported younger age of onset in females (second and third decades) than in males (third and fourth decades). 3 4

In pediatric patients, MG has the following three distinct subcategories that have variable pathophysiology: (1) congenital myasthenic syndrome, (2) transient neonatal myasthenia, and (3) juvenile MG. 5 The diagnosis of MG is based on the clinical presentation with skeletal muscle weakness which improves after a period of rest. 6 However, multiple diagnostic modalities can be used to confirm the diagnosis, such as ice pack and electrodiagnostic tests, which include repetitive nerve stimulation (RNS) and single-fiber electromyography (SFEMG). Yet the presence of serum AChR antibodies is the first-line investigation. 7 Treatment of MG is variable and depends on several factors, such as the patient's age, severity, and disease distribution. The treatment modalities are anticholinesterase drugs, immunosuppressive drugs, corticosteroids, surgical thymectomy, plasma exchange (PLEX), and intravenous immunoglobulin (IVIg). Despite the variability of treatment modalities, the aim of all of these therapies is to restore normal muscle strength. 8

In dentistry, providing routine dental treatment of MG patients is challenging and requires special considerations and awareness about the condition's complexity from the dental team. Review of the patient's medical history and consultation from the treating physician is mandatory prior to any dental treatment. Particularly, in the use of local anesthesia, ester local anesthetic is inactivated by plasma cholinesterase, so in patients with MG receiving anticholinesterase medication, the effect of local anesthesia will be prolonged, leading to toxicity; hence, amide local anesthesia is preferable. 6 In addition, several medications are contraindicated such as aminoglycosides and fluoroquinolones. Lastly, providing convenient, early morning appointments and a stress-free environment are recommended to prevent a MG crisis. 6

In this case report, we present the case of a school-aged girl diagnosed with MG where full-mouth dental rehabilitation under general anesthesia was performed. Owing to the rareness of pediatric MG, our aim is to raise awareness about MG among dentists and to discuss the dental management and precautions that need to be taken prior to dental procedures.

## Case Report

A school-aged Saudi girl was referred from her neurologist as a known case of MG for the treatment of multiple dental caries. Prior to her diagnosis, the patient frequently complained of ataxia and generalized muscle weakness. She was previously admitted with aspiration pneumonia during which she had two cardiac arrests requiring intubation for 2 weeks. Subsequently, multiple admissions were done for comprehensive workup, then the diagnosis was made by a neurologist at King Abdullah Specialized Children Hospital, Riyadh, Saudi Arabia, in December 2018 when the patient was at the age of 5 years.

Moreover, the patient had other comorbidities. She had iron-deficiency anemia and high blood pressure; nevertheless, her antihypertensive medications were recently discontinued in April 2020 by her treating physician. She also had a history of resolved posterior reversible encephalophagy syndrome and grade-1 hydronephrosis of the right kidney. Her MG was treated with pyridostigmine, azathioprine, plasmapheresis (PLEX), and IVIg. However, the patient suffered from a crisis after IVIg infusion and needed to be admitted on multiple occasions. In December 2019, a thymectomy was performed, and the patient was currently on pyridostigmine 60-mg q6 hour only. She is allergic to 38 medications which include but are not limited to penicillin, erythromycin, clindamycin, sulfa drugs, and lidocaine.

**Fig. 1:**
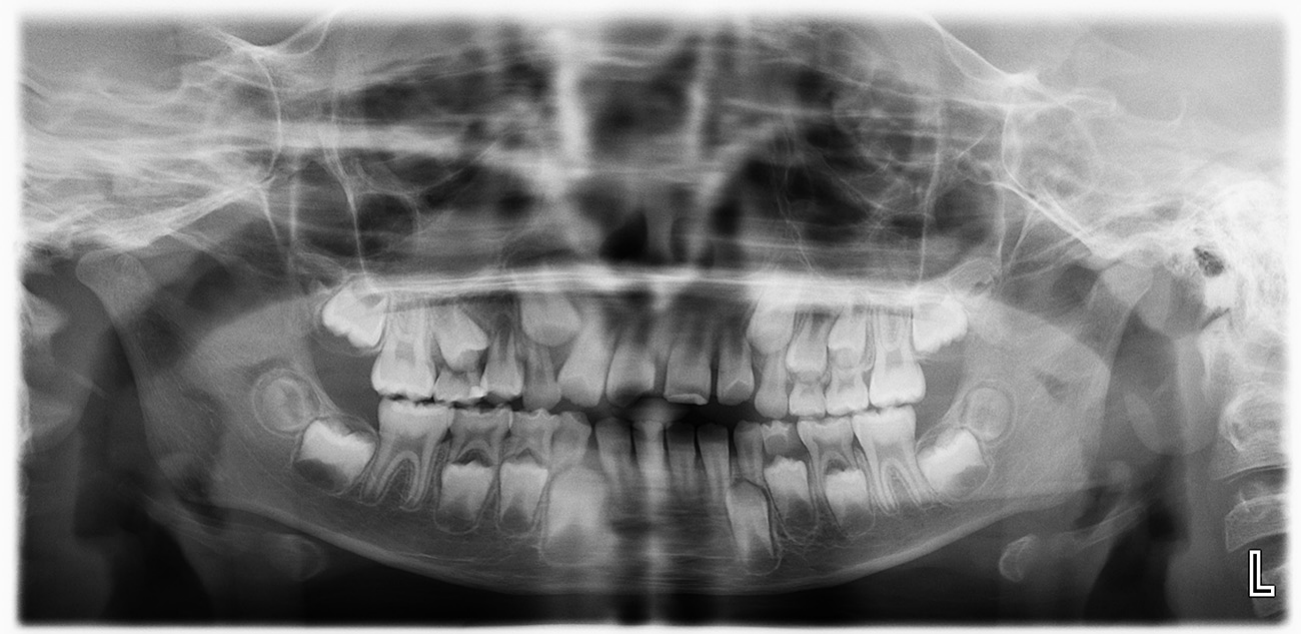
Preoperative panoramic view radiograph.

**Fig. 2:**
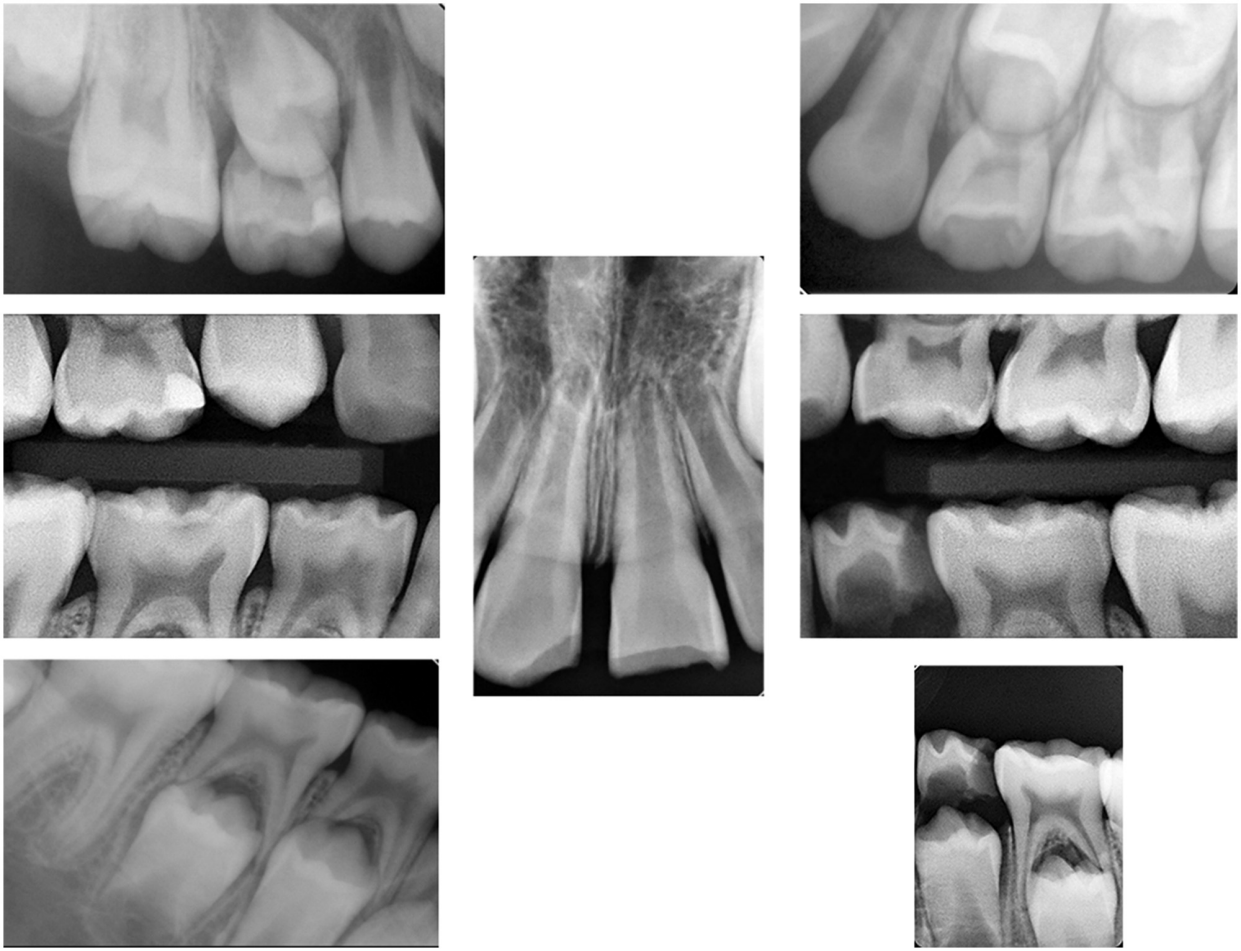
Preoperative intraoral radiographs.

**Fig. 3:**
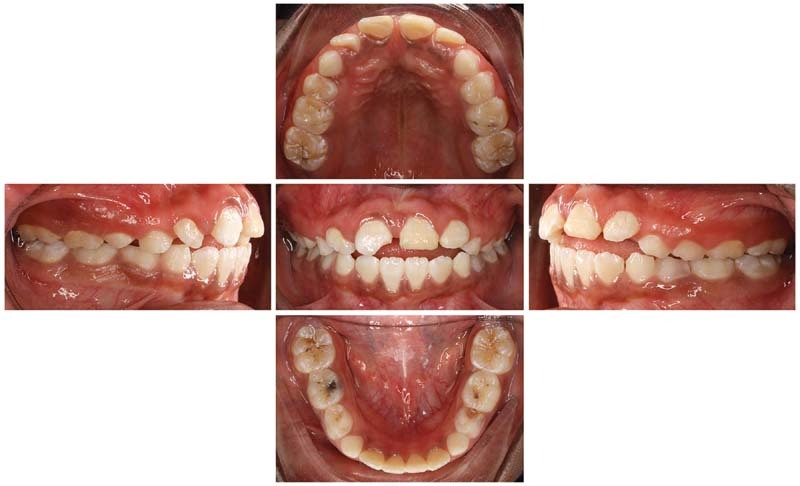
Preoperative intraoral photographs.

Oral examination showed poor oral hygiene, a mixed dentition stage, fluorosis manifested as generalized white spots affecting all dentition, multiple dental caries, tendency to anterior open-bite due to a tongue thrusting habit, and an uncomplicated enamel\dentin crown fracture of the upper permanent central incisors which occurred 3 weeks ago and was treated temporarily with resin-modified class ionomer restoration. Panoramic views ( Fig. 1 ), bitewings and periapical radiographs ( Fig. 2 ), and preoperative intraoral photographs ( Fig. 3 ) were taken. The radiographs revealed normal developing dentition and multiple occlusal and proximal caries with signs of periapical radiolucency related to the upper right primary second molar. After case assessment, the decision was made by the dental team to treat the patient under general anesthesia owing to her medical condition, multiple allergies, and the extent of the dental treatment needed. In regard to the tongue thrusting habit, orthodontic consultation was obtained where the recommendation was to not intervene at this stage and to reevaluate after 6 months. Furthermore, consultations were performed with the neurology, nephrology, and anesthesia departments for clearance. The neurologist considered her to be a high-risk patient and recommended admission for 1 day postoperatively for monitoring in a high-dependency unit (HDU), where the anesthesiologist cleared her as American Society of Anesthesiology (ASA) III. Informed consent was obtained from the patient's father regarding the full dental rehabilitation under general anesthesia and the publication of a case report including the sharing of the patient's medical history, radiographs, and intraoral photographs.

On the day of the surgery, the patient was brought into the operating room, nasal intubation was performed by the anesthesia team, and time-out was declared by the dental team. Full-mouth dental rehabilitation was performed which included pit and fissure sealant for tooth no. 14; class-I composite restorations for teeth nos. 16 and 46; class-IV composite restorations for teeth nos. 11 and 21; preventive resin restorations for teeth nos. 65, 26, and 36; stainless steel crowns (SSCs) for teeth nos. 64, 75, and 84; indirect pulp capping and SSCs for tooth no. 85; and the extraction of teeth nos. 55 and 74. The patient was then admitted for 1 day and was discharged the next day without any complications.

**Fig. 4:**
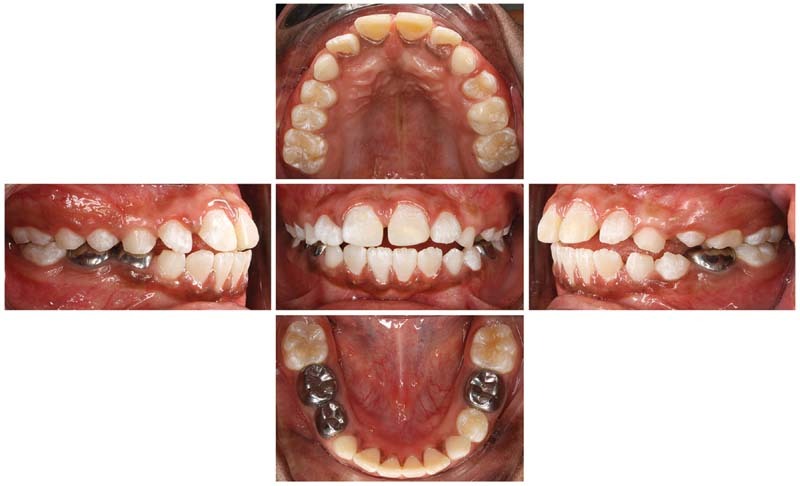
Recall intraoral photographs at 6 months.

**Fig. 5:**
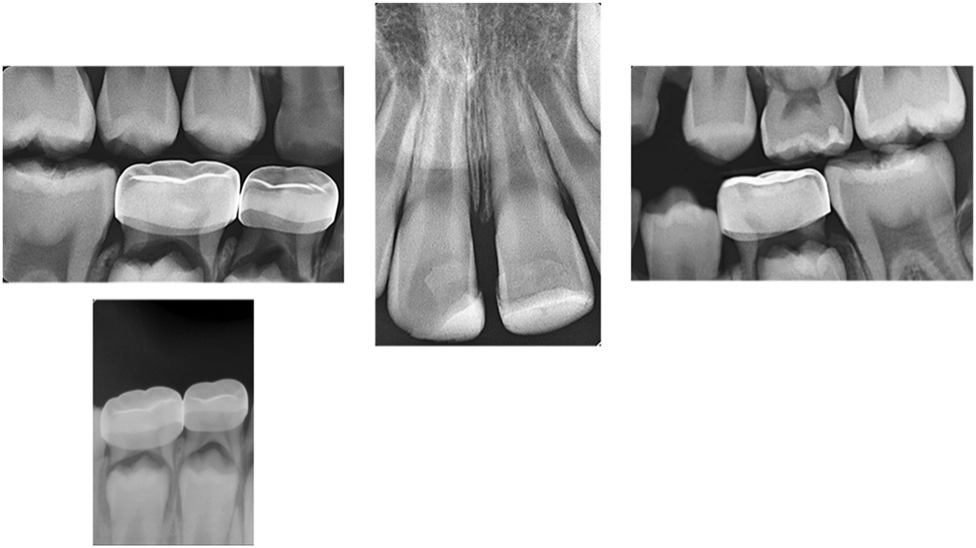
Recall intraoral radiographs at 6 months.

**Fig. 6:**
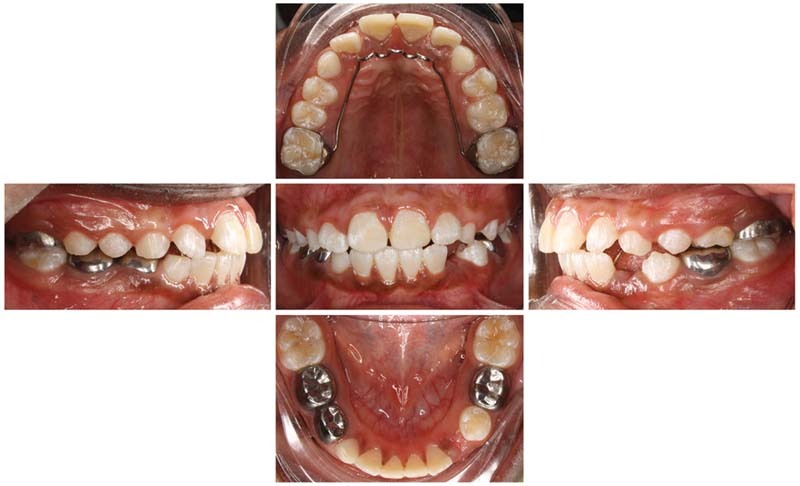
Postpalatal crib appliance placement intraoral photographs at 1 month.

Follow-up appointments were scheduled at 2 weeks, 6 weeks, 3 months, and 6 months. The 6-week visit was to evaluate the traumatized upper permanent central incisors and to take periapical radiographs as recommended by the American Academy of Pediatric Dentistry guidelines. The clinical examination revealed no signs of increased mobility, discoloration, or dental abscesses, and the radiographic examination showed no sign of periapical radiolucency. At the 3-month recall, the patient presented with improved oral hygiene, intact restorations and crowns, erupting premolars, and no new carious lesions. Furthermore, at the 6-month recall, intraoral photographs ( Fig. 4 ), radiographs ( Fig. 5 ), and orthodontic consultations were performed. The clinical and radiograph examinations revealed no signs of new caries or periapical lesions, while the updated orthodontic consultation including the placement of a palatal crib appliance for 6 months to stop the tongue thrusting habit and there was a noticeable improvement after 1 month ( Fig. 6 ). The patient will be seen routinely every 3 months, and orthodontic consultation will be updated accordingly.

## Discussion

MG can be classified based on the autoantibodies present, the age of the patient at onset, the location of muscle weakness, and the thymic abnormalities. 9 10 The majority of MG cases start as the pure ocular form where patients present with only a weakness of the extraocular muscles, leading to ptosis and diplopia. However, 80 to 85% of cases progress to the generalized form which involves but is limited to weakness in the facial, masticatory, soft palatal, and proximal limb muscles. As a result, these patients usually have dysphagia, dysarthria, expressionless faces, nasal speech, and difficulties in movement. 6 9 The severity of the muscle weakness tends to worsen as the day progresses. Moreover, stresses, infection, hormonal changes, temperature changes, and medications that work on neuromuscular transmission can affect the myasthenic symptoms. 11 In our case, the patient had a generalized form with anti-AChR antibodies, and she suffered from ptosis, ataxia, and generalized proximal limb weakness.

Treatment of MG is case dependent and involves multiple factors, such as the age of the patient, the degree of severity, the distribution of muscle weakness, and the presence of comorbidities. 8 Anticholinesterase drugs are the first-line treatment, and they inhibit the breakdown of acetylcholine. Oral corticosteroids are commonly used, and there is a significant improvement in approximately 80% of patients that take corticosteroids. In addition, immunosuppressive medications such as azathioprine and cyclosporine are used but with common side effects such as leukopenia, pancytopenia, and infection. 6 8 In cases of MG crisis or when patients need to receive rapid treatment, PLEX and IVIg are chosen. 6 In this case, our patient received all of the above mentioned medications. However, she is currently on anticholinesterase and ferrous sulfate only. Her medical condition improved after the thymectomy.

A myasthenic crisis is considered a life-threatening event where patients need immediate clinical attention. 6 MG is characterized by acute respiratory insufficiency and could be triggered by infections, surgical procedures, drugs, and emotional stress. 12 13 The prevalence of myasthenic crisis is approximately 15 to 20%, where 4 to 8% of cases are fatal. 6 In our case, she had two myasthenic crises where she had to be admitted to the pediatric intensive care unit (PICU). However, she did not experience any myasthenic crisis after the thymectomy. In fact, studies showed that 85% of patients with MG improved after thymectomy, despite the presence of thymus abnormalities which are similar to this case. 12

Dental treatment is known to be a stressful procedure for several reasons, such as the fear of pain, the fear of injection, and the sound and sight of the dental equipment. In MG patients, stress is considered a risk factor that can trigger a myasthenic crisis and needs to be reduced throughout the appointment. Short, early appointments are recommended, and oral acetylcholine should be taken approximately 1 hour before the dental appointment to benefit from having a maximal muscle strength and to avoid fatigue. 14 15 In fact, consultation with the primary care physician is crucial for the safety of using certain medications that can have synergistic toxic effects or predispose to a myasthenic crisis or if there is a need for prophylactic antibiotics or any other precautions that need to be taken. Amide local anesthetics should be used because ester type local anesthetics may show a long-lasting effect and have an increased risk of systemic toxicity in patients receiving anticholinesterase drugs. 6 Nitrous oxide sedation and general anesthesia are safe considering the importance of airway patency. 16 17 In our patient, general anesthesia was chosen given the behavioral difficulties, the comprehensive dental treatment needed, and the primary physician's recommendation because the patient was considered high risk and hospital admission was needed.

## Conclusion

MG is a rare medical condition with a variable clinical presentation. A detailed medical history, dental history, and physician consultation are mandatory before any dental procedure. Dentists should educate the families of the patient regarding the importance of maintaining good oral hygiene to reduce the risks of having invasive dental procedures.
